# Reactive Oxygen Species Regulate Caspase-11 Expression and Activation of the Non-canonical NLRP3 Inflammasome during Enteric Pathogen Infection

**DOI:** 10.1371/journal.ppat.1004410

**Published:** 2014-09-25

**Authors:** Christopher R. Lupfer, Paras K. Anand, Zhiping Liu, Kate L. Stokes, Peter Vogel, Mohamed Lamkanfi, Thirumala-Devi Kanneganti

**Affiliations:** 1 Department of Immunology, St. Jude Children's Research Hospital, Memphis, Tennessee, United States of America; 2 Veterinary Pathology Department, St. Jude Children's Research Hospital, Memphis, Tennessee, United States of America; 3 Department of Medical Protein Research, Vlaams Instituut voor Biotechnologie, Ghent, Belgium; 4 Department of Biochemistry, Ghent University, Ghent, Belgium; Stanford University School of Medicine, United States of America

## Abstract

Enteropathogenic and enterohemorrhagic bacterial infections in humans are a severe cause of morbidity and mortality. Although NOD-like receptors (NLRs) NOD2 and NLRP3 have important roles in the generation of protective immune responses to enteric pathogens, whether there is crosstalk among NLRs to regulate immune signaling is not known. Here, we show that mice and macrophages deficient in NOD2, or the downstream adaptor RIP2, have enhanced NLRP3- and caspases-11-dependent non-canonical inflammasome activation in a mouse model of enteropathogenic *Citrobacter rodentium* infection. Mechanistically, NOD2 and RIP2 regulate reactive oxygen species (ROS) production. Increased ROS in *Rip2*-deficient macrophages subsequently enhances c-Jun N-terminal kinase (JNK) signaling resulting in increased caspase-11 expression and activation, and more non-canonical NLRP3-dependant inflammasome activation. Intriguingly, this leads to protection of the colon epithelium for up to 10 days in *Rip2*-deficient mice infected with *C. rodentium*. Our findings designate NOD2 and RIP2 as key regulators of cellular ROS homeostasis and demonstrate for the first time that ROS regulates caspase-11 expression and non-canonical NLRP3 inflammasome activation through the JNK pathway.

## Author Summary

Caspase-11 is required for NLRP3 inflammasome activation and cell death in response to certain gram-negative bacterial infections like *Citrobacter rodentium*. However, how *C. rodentium* drives caspase-11 expression and activation is not well understood. Here, we demonstrate that the NOD2-RIP2 pathway regulates reactive oxygen species production and c-Jun N-terminal kinase signaling to control caspase-11 expression and subsequent activation of caspase-11 and the NLRP3 inflammasome during *C. rodentium* infection. In the absence of NOD2-RIP2 signaling, increased inflammasome activation results in lower bacteria numbers in the colon and less tissue damage during the early stages of infection.

## Introduction

Enteropathogenic and enterohemorrhagic *Escherichia coli* (EPEC and EHEC) infections in humans are a major source of morbidity and mortality, especially in developing countries [1]. To study these infections, *Citrobacter rodentium* is used as an enteric bacterial pathogen of mice that triggers similar inflammatory responses to those observed in humans infected with EPEC and EHEC [2]. As with EPEC and EHEC in humans, *C. rodentium* infection in the mouse results in bacterial attachment and effacing lesion formation in the lumen of the colon [3]. Clearance of *C. rodentium* from the host tissues requires contributions from humoral and Th1 immune responses [4]–[6]. However, innate immunity is also important for early bacterial control [7], [8] and for the initiation of adaptive immunity [9], [10].

Innate immunity to pathogens depends on a limited set of germ-line encoded pattern recognition receptors (PRRs) that sense conserved pathogen motifs. Innate receptors initiate inflammation through activation of pro-inflammatory transcription factors such as NF-κB, by promoting activation of proinflammatory caspases in inflammasomes, and by initiating programmed cell death of infected cells. The intracellular NOD-like receptors (NLRs) NOD1 and NOD2 recognize peptidoglycan fragments from bacterial cell walls in the cytosol, which results in pro-inflammatory NF-κB and MAP-kinase pathway activation via the adaptor RIP2 [11]–[13]. However, pathogen infection is frequently accompanied by ion fluxes and vacuolar membrane damage elicited by the action of microbial toxins and effectors of specialized bacterial secretion systems [14]–[17]. Thus, while conserved bacterial peptidoglycan fragments are initially recognized by receptors NOD1 and NOD2, the perturbations inflicted over the course of infection can activate NLRP3.

NLRP3 is the most studied inflammasome-associated NLR. Signaling events initiated by Toll-like receptors (TLRs) contribute to NF-κB mediated up-regulation of NLRP3 and pro-IL-1β for robust activation [18]. No specific pathogen-derived ligand is known to bind and activate NLRP3. Instead, NLRP3 is activated in response to diverse stimuli including microbial, environmental and metabolic perturbations, which culminate in the generation of reactive oxygen species, changes in ion flux or leakage of cathepsin B into the cytoplasm [19]–[21]. These cellular damage signals induce NLRP3 to form an inflammasome complex with the adaptor ASC and the cysteine protease caspase-1 that enables the maturation of IL-1β and IL-18 cytokines and pyroptotic cell death [22]–[25]. An alternative non-canonical pathway of NLRP3 activation that additionally involves upstream caspase-11 activation has been described in response to cytosolic LPS and gram-negative enteric pathogens such as *Citrobacter rodentium* and *Escherichia coli* [26], [27]. Recent studies have extended the requirement of the non-canonical inflammasome to other gram-negative pathogens such as flagellin-deficient *Legionella pneumophila (ΔflaA)* [28] and *Salmonella typhimurium (Δfljb/flic)* [29]. Studies from our lab and others suggest that activation of the non-canonical NLRP3 inflammasome requires TLR4-TRIF mediated type-I interferon dependent production of caspase-11 [30], [31]. However, whether NLR signaling pathways can regulate the non-canonical NLRP3 inflammasome is not known. Here, we show that activation of the cytoplasmic NOD2-RIP2 pathway modulates caspases-11 expression and activation of the non-canonical inflammasome in response to *C. rodentium* and *Δfljb/flic Salmonella typhimurium* infection.

## Results

### *Nod2*- and *Rip2*-deficient cells display enhanced inflammasome activation

**Figure 1:**
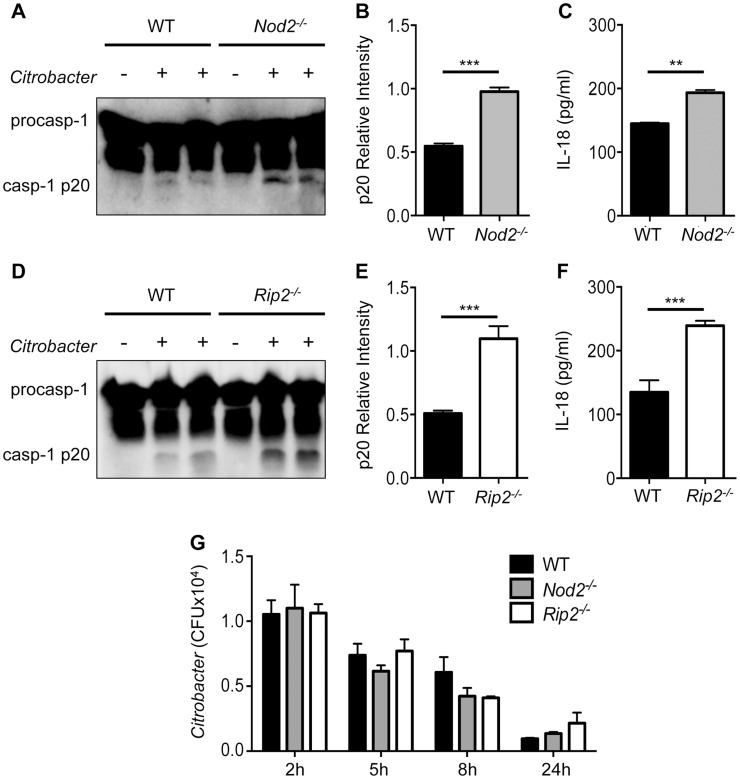
Enhanced inflammasome activation in *Nod2^−/−^* and *Rip2^−/−^* BMDMs. BMDM were generated from WT, *Nod2^−/−^* and *Rip2^−/−^* mice and infected with 20MOI of *Citrobacter rodentium* for 18 h. **(A–F)** Combined supernatant and lysates were examined by Western blot for caspase-1 cleavage (casp-1p20) visually (A,D) and by densitometry (B,E), or (C,F) supernatants were examined for IL-18 secretion by ELISA. **(G)** BMDM were infected with *C. rodentium* and assayed for intracellular growth at the indicated times post-infection. (A–F) Data are representative of five independent experiments with n = 2–3 wells per experiment. (G) Data are representative of two independent experiments with n = 2–3 wells per experiment. (B,C,E,F,G) Data are shown as the mean ± SEM. (*, p<0.05; **, p<0.01; ***, p<0.001).

Signaling events initiated by TLRs contribute to NF-κB mediated up-regulation of NLRP3 and pro-IL-1β [18]. However, whether TLR or NLR pathways can restrain or negatively regulate inflammasome activation is not known. To examine the role of NOD2 in inflammasome activation, WT and *Nod2^−/−^* bone marrow derived macrophages (BMDM) were infected with *C. rodentium* and caspase-1 activation was analyzed at 18 h post-infection. *Nod2*-deficient cells exhibited increased caspase-1 activation and this was accompanied with elevated production of IL-18 (**Figure 1A–C**). Similarly, *Rip2^−/−^* cells displayed increased caspase-1 activation and IL-18 secretion (**Figure 1D–F**). Furthermore, increased caspase-1 activation was not due to increased bacterial loads because *Nod2^−/−^* and *Rip2^−/−^* macrophages exhibited comparable *C. rodentium* uptake and clearance (**Fig. 1G**). These results suggest that the NOD2-RIP2 axis negatively regulates inflammasome activation to *C. rodentium* infection.

### The NOD2-RIP2 axis specifically restricts the NLRP3 inflammasome

**Figure 2:**
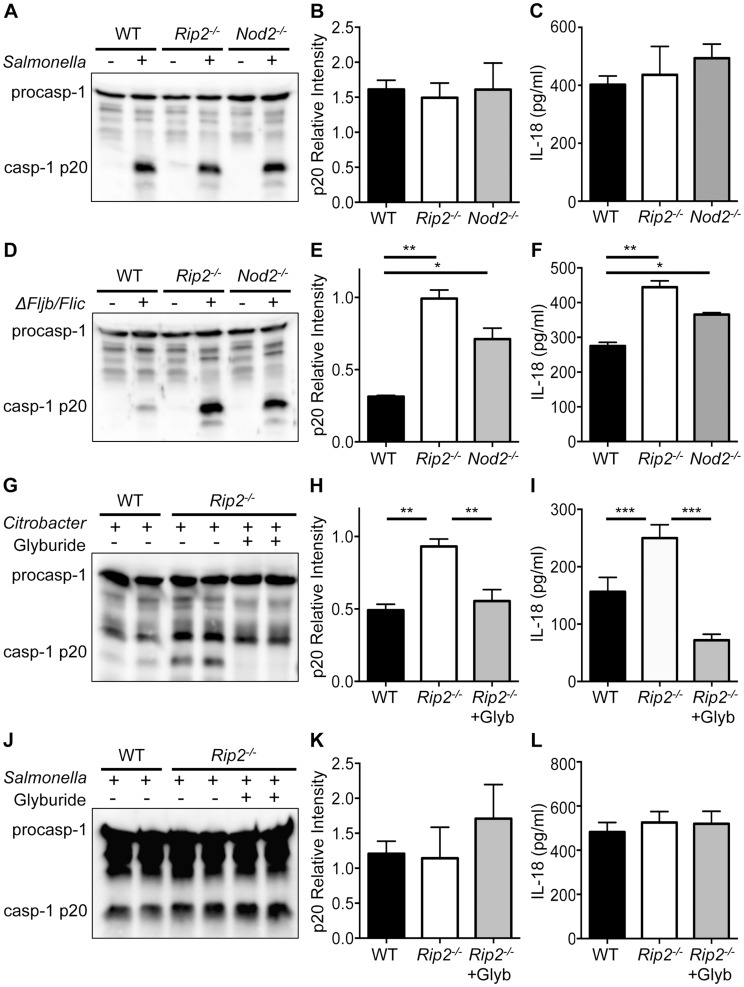
NOD2 and RIP2 specifically regulate NLRP3 inflammasome activation. **(A–B)** WT, *Nod2^−/−^* and *Rip2^−/−^* BMDM were infected with 1MOI *Salmonella typhimurium* for 4 h and combined supernatant and lysates were examined by Western blot for caspase-1 cleavage (casp-1p20) visually (A) and by densitometry (B). **(C)** Supernatants were examined 4 h after *S. typhimurium* infection for IL-18 secretion. **(D–F)** WT, *Nod2^−/−^* and *Rip2^−/−^* BMDM were infected with 10MOI *S typhimurium Δfljb/flic* mutant for 18 h. Caspase-1 activation and IL-18 were examined as in panels A–C. **(G–I)** WT and *Rip2^−/−^* BMDM were infected with 20MOI of *C. rodentium* for 2 h and *Rip2^−/−^* cells were subsequently treated with the NLRP3 specific inhibitor glyburide or mock treated as a control. Caspase-1 activation and IL-18 were examined as in panels A–C. **(J–L)** WT and *Rip2^−/−^* BMDM were infected with 1MOI of *S. typhimurium* and *Rip2^−/−^* cells were simultaneously treated with the NLRP3 specific inhibitor glyburide or mock treated as a control. Caspase-1 activation and IL-18 were examined as in panels A–C. (A–L) Data are representative of three independent experiments with n = 2–3 wells per experiment and displayed as the mean ± SEM. (*, p<0.05; **, p<0.01; ***, p<0.001).

Activation of caspase-1 is triggered downstream of several NLRs including NLRP3, NLRP1 and NLRC4 [23], [32], [33]. Previously, we have shown that infection with *C. rodentium* specifically activates the non-canonical NLRP3 inflammasome [30]. To determine the extent of NOD2-RIP2 modulation, we examined whether this pathway also modulates the NLRC4 inflammasome. To answer this, we infected WT, *Nod2*- and *Rip2*-deficient cells with *Salmonella typhimurium* to activate the NLRC4-inflammasome. However, comparable caspase-1 activation was observed in *S. typhimurium* infected WT, *Nod2^−/−^* and *Rip2^−/−^* cells (**Figure 2A,B**). In agreement, IL-18 levels were similar (**Figure 2C**) suggesting that the NOD2-RIP2 pathway specifically restricts the NLRP3 inflammasome. The NLRC4 inflammasome is activated upon recognition of *S. typhimurium* flagellin [34], [35]. However, flagellin-deficient *S. typhimurium Δfljb/flic* activates the NLRP3 inflammasome [29]. Consequently, *Nod2^−/−^* or *Rip2^−/−^* BMDM infected with the *S. typhimurium Δfljb/flic* strain displayed enhanced caspase-1 activation and IL-18 production over wildtype BMDM (**Figure 2D–F**). These results suggest that NOD2 and RIP2 specifically modulated the NLRP3 inflammasome in this model while the NLRC4 inflammasome was not affected by the absence of the NOD2-RIP2 signaling axis.

Glyburide, a type 2 diabetes drug, has been shown to specifically inhibit the NLRP3 inflammasome in response to microbial and crystalline stimuli [36]. To further verify that the enhanced caspase-1 activation observed in *Nod2*- and *Rip2*-deficient BMDM is the result of elevated NLRP3 inflammasome activation, we exposed *Rip2*-deficient BMDM to glyburide following *C. rodentium* infection. Consistent with the requirement for NLRP3, we observed increased caspase-1 activation in *Rip2*-deficient cells and this was markedly abrogated in the presence of glyburide (**Figure 2G,H**). In agreement, enhanced IL-18 levels in *Rip2^−/−^* BMDM were significantly decreased upon treatment with glyburide (**Figure 2I**). In contrast, glyburide treatment did not affect caspase-1 activation in *Rip2*-deficient cells infected with *S. typhimurium* (**Figure 2J,K**). Consequently, IL-18 levels were also unaffected (**Figure 2L**). These data indicate that NOD2-RIP2 signaling specifically modulates NLRP3 activation and does not regulate all inflammasomes.

### *Rip2*-deficient cells show increased ROS production

**Figure 3:**
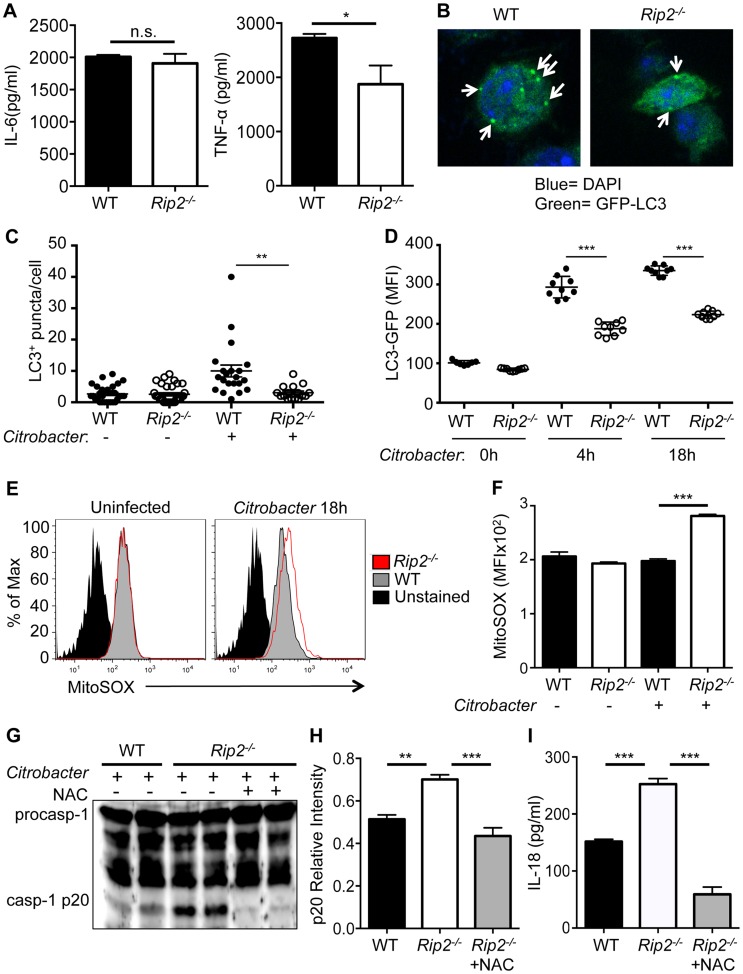
Increased mitochondrial damage in *Rip2^−/−^* cells drives enhanced inflammasome activation. WT and *Rip2^−/−^* BMDM were infected with 20MOI of *C. rodentium* for 18 h. **(A)** Supernatants were collected and analyzed for IL-6 and TNF-α secretion. **(B–D)** WT-LC3-GFP and *Rip2^−/−^*-LC3-GFP BMDM were infected with 20MOI of *C. rodentium* and were examined for LC3^+^ autophagosomes (Arrows) at 18 h by confocal microscopy (B–C) or LC3 geometric mean fluorescence intensity (MFI) at 4 h and 18 h by flow cytometry (D). **(E–F)** BMDM were stained with the mitochondrial specific superoxide sensitive dye MitoSOX and analyzed by flow cytometry. **(G–H)** WT and *Rip2^−/−^* BMDM were infected with 20MOI of *C. rodentium* for 2 h and *Rip2^−/−^* cells were subsequently treated with NAC or mock treated as a control. After 18 h, supernatant and lysates were combined and examined by Western blot for caspase-1 cleavage (casp-1p20) visually (G) and by densitometry (H). **(I)** Supernatants were also examined at 18 h after *C. rodentium* infection for IL-18 secretion. (A,E–I) Data are representative of three independent experiments with n = 2–4 wells per experiment. (B–C) Data are representative of 2–3 independent experiments with n = 1–3 wells per experiment and displayed as the mean ± SEM. (*, p<0.05; **, p<0.01; ***, p<0.001).

To determine the cause of increased NLRP3 inflammasome activation during *C. rodentium* infection, we examined the possibility that there is a global dysregulation of cytokines and inflammation in *Rip2*-deficient BMDM. However, there was no difference in IL-6 production and TNF-α levels were lower in *Rip2*-deficient cells (**Figure 3A**) suggesting that a global increase in inflammation is not responsible for increased NLRP3 inflammasome activation in this model.

Elevated production of reactive oxygen species (ROS) has previously been associated with increased NLRP3 inflammasome activation [37]. In particular, mitochondrial-derived ROS production was suggested to provoke NLRP3 activation [38], and we previously demonstrated that *Rip2*-deficient cells have defects in autophagy that lead to the accumulation of damaged mitochondria [39]. LC3 is an autophagy-associated protein that is localized in the cytosol under steady state conditions, but relocalizes to autophagosome membranes when autophagy occurs [40]. During *C. rodentium* infection of WT or *Rip2^−/−^* BMDM, the number of autophagosomes was examined using confocal microscopy by counting GFP-LC3^+^ puncta per cell (**Figure 3B–C**). Alternatively, we used flow cytometry to measure the fluorescence intensity of GFP-LC3 after permeablizing cells so that only autophagosome membrane bound GFP-LC3 remained (**Figure 3D**). In both cases, we found that autophagy was impaired in *Rip2^−/−^* BMDM compared to WT cells after *C. rodentium* infection. Furthermore, *Rip2*-deficient BMDM exhibited elevated levels of mitochondria-derived superoxide when infected with *C. rodentium* and stained with the mitochondrial specific ROS sensor MitoSOX (**Figure 3E,F**). Finally, *Rip2^−/−^* BMDM treated with the ROS scavenger N-acetyl-L-cysteine (NAC) had reduced caspase-1 activation and IL-18 production compared to WT cells (**Figure 3G–I**). Thus, the presence of dysfunctional mitochondria in *Rip2^−/−^* cells leads to enhanced ROS production and thereby increased NLRP3 inflammasome activation.

### Caspase-11 activation is upregulated in *Rip2*-deficient cells

**Figure 4:**
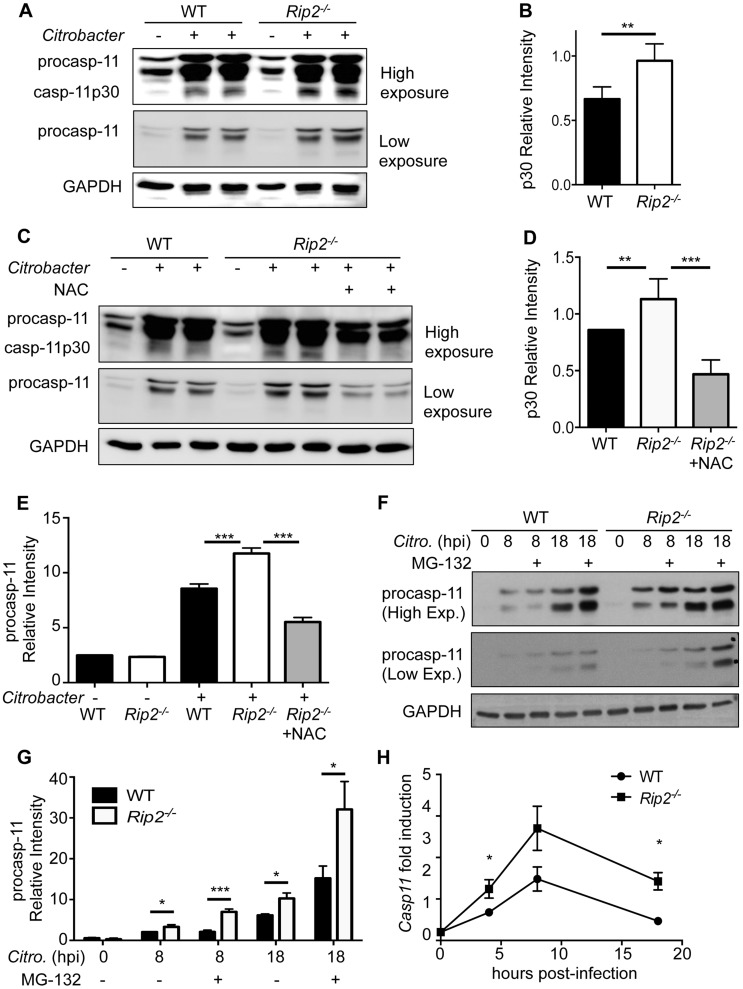
RIP2 regulates the caspase-11 non-canonical inflammasome. **(A–B)** WT and *Rip2^−/−^* BMDM were infected with 20MOI of *C. rodentium* for 18 h and combined supernatant and lysates were examined by Western blot for caspase-11 cleavage (casp-11p30) visually (A) and by densitometry (B). **(C–D)** WT and *Rip2^−/−^* BMDM were infected with 20MOI of *C. rodentium* for 2 h and *Rip2^−/−^* cells were subsequently treated with NAC or mock treated as a control. Samples were collected at 18 h and caspase-11 activation was examined as in panels A–B. **(E)** Densitometry was performed on pro-Caspase-11 p43 band from 3 independent experiments. **(F–G)** WT and *Rip2^−/−^* BMDM were infected with 20MOI of *C. rodentium* for 4 h and cells were subsequently treated with 1 µM MG-132 or mock treated as a control. Samples were collected at the indicated time points and pro-caspase-11 levels examined by Western blot and densitometry. **(H)** WT and *Rip2^−/−^* BMDM were infected with 20MOI of *C. rodentium* and RNA collected at the indicated time points and analyzed by qRT-PCR for fold induction and normalized to GAPDH. (A–H) Data are representative of three independent experiments with n = 2–3 wells per experiment and displayed as the mean ± SEM. (**, p<0.01; ***, p<0.001).

Enteric pathogens such as *C. rodentium* are known to activate the NLRP3 inflammasome by a non-canonical pathway, which additionally requires caspase-11 for its activation [26]. We therefore postulated that during *C. rodentium* infection increased ROS production might act upstream of caspase-11 as well as caspase-1. Therefore, we sought to examine the role of caspase-11 in the present study. First, we determined that activation of caspase-11 is enhanced in *Rip2^−/−^* BMDM (**Fig. 4A,B**). Next, we treated *Rip2^−/−^* BMDM with the ROS scavenger NAC following *C. rodentium* infection and observed that caspase-11 activation was significantly diminished compared to mock-treated *Rip2^−/−^* BMDM controls (**Figure 4C,D**). We also found that caspase-11 activation (p30 band) correlated with the expression of p43/p38 (pro-caspase-11). Indeed, densitometric analysis showed that *C. rodentium* infected *Rip2^−/−^* BMDM had higher expression of pro-caspase-11 and that treatment with NAC inhibited pro-caspase-11 expression (**Figure 4E**). Mechanistically, we examined whether the increased caspase-11 seen in *Rip2^−/−^* BMDM was the result of defects in protein turnover; however, treatment of WT and *Rip2^−/−^* BMDM 4 h after infection with the proteasome inhibitor MG-132 resulted in accumulation of caspase-11 in both WT and *Rip2^−/−^* BMDM (**Figure 4F–G**). Instead, *Casp11* mRNA was higher in *Rip2^−/−^* BMDM compared to WT BMDM (**Figure 4H**). These results indicate that RIP2 regulates caspase-11 at the level of mRNA expression, possibly through enhanced ROS production.

### Exogenous ROS enhances caspase-11 expression and activation in WT cells

**Figure 5:**
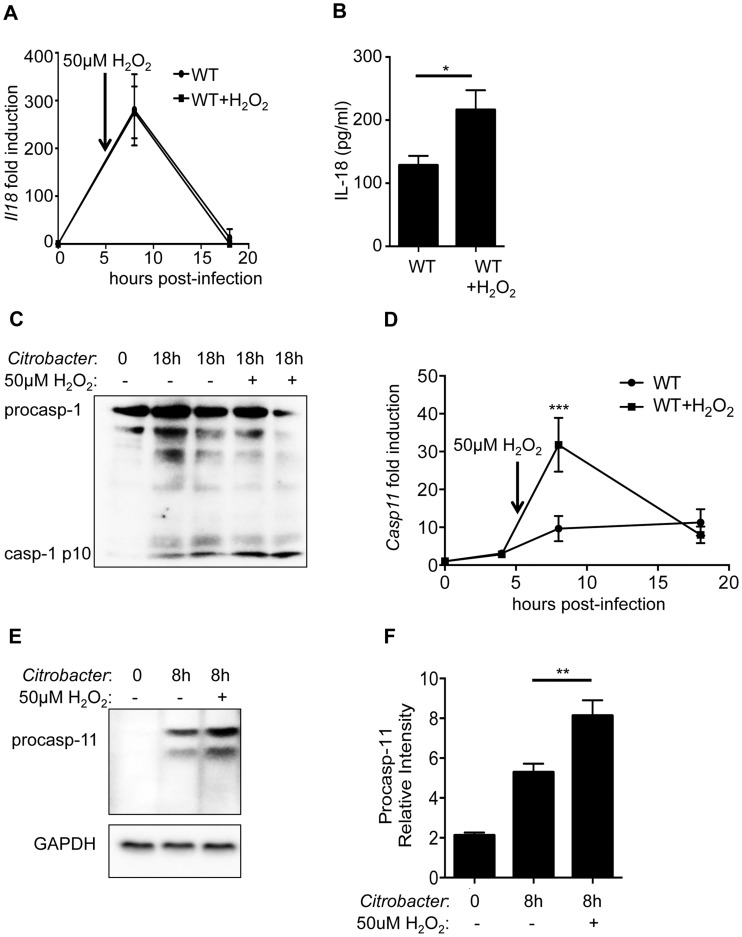
ROS enhances caspase-11 expression. WT BMDM were infected with 20MOI of *C. rodentium* for 6 h and mock treated or treated with 50 µM of H_2_O_2_ as a ROS source. **(A)** BMDM were collected at 8 h or 18 h after initial infection and examined for *Il18* mRNA expression by qRT-PCR. **(B)** Supernatants were collected 18 h post-infection and IL-18 levels determined by ELISA. **(C)** WT BMDMs were examined by Western blot for caspase-1 activation. **(D–F)** Caspase-11 expression was examined at the (D) mRNA level by qRT-PCR or the (E–F) protein level by Western blot and densitometric analysis. (A–F) Data are representative of three independent experiments with n = 2–4 wells per experiment and displayed as the mean ± SD. (*, p<0.05; ***, p<0.001).

To further confirm the role of enhanced ROS production leading to increased inflammasome activation in *Rip2^−/−^* BMDM, we examined the effects of treating WT BMDM with exogenous ROS. WT BMDM were infected with *C. rodentium* and treated with H_2_O_2_ as a ROS source 6 h after infection. Although H_2_O_2_ treatment had no effect on *Il18* mRNA levels following *C. rodentium* infection, we detected significantly more IL-18 in the culture medium (**Figure 5A,B**). In agreement, caspase-1 activation was also increased following H_2_O_2_ treatment (**Figure 5C**). Importantly, H_2_O_2_ treatment of WT cells resulted in increased mRNA and protein expression of caspase-11 by qRT-PCR and Western blot respectively (**Figure 5D–F**). Our results indicate that ROS produced during *C. rodentium* infection can enhance caspase-11 expression and subsequently potentiate activation of the non-canonical inflammasome.

### ROS regulates caspases-11 through a JNK mediated pathway

**Figure 6:**
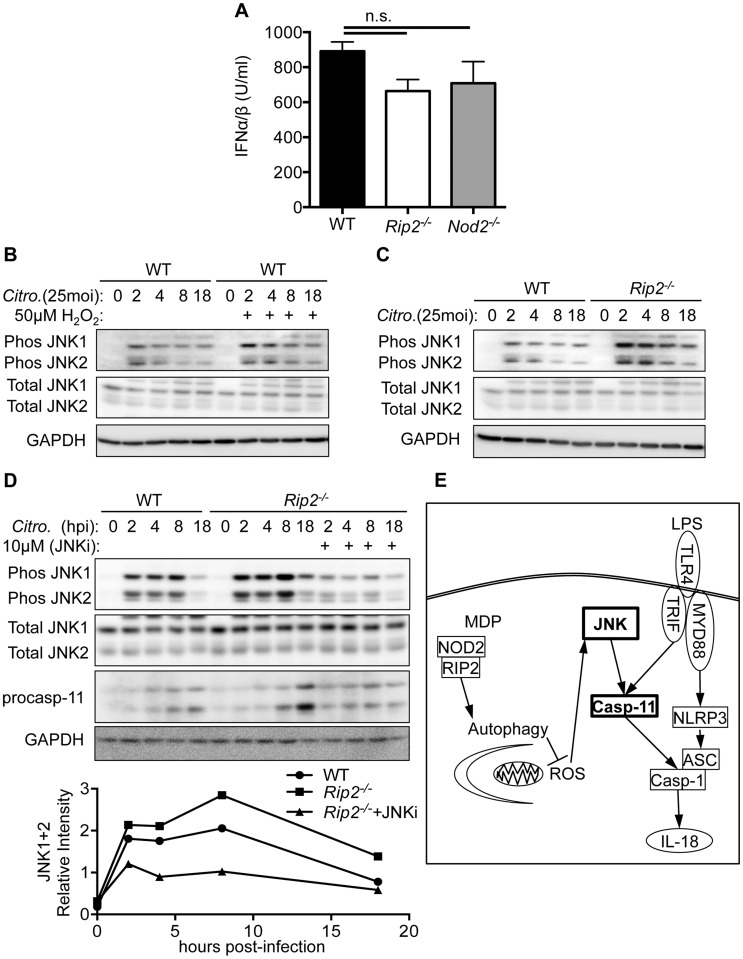
RIP2 regulates caspases-11 expression through a ROS-JNK pathway. **(A)** WT and *Rip2^−/−^* BMDM were infected with 20MOI of *C. rodentium* for 18 h and supernatants collected and examined for type-I interferon using a reporter cell line (U = Units). **(B–C)** WT or *Rip2^−/−^* BMDM were infected with 20MOI of *C. rodentium* and mock treated or treated with H_2_O_2_. Samples were collected at the indicated times and examined for total JNK or phosphorylated JNK by Western blot. **(D)** WT or *Rip2^−/−^* BMDM were infected with 20MOI of *C. rodentium* and mock treated or treated with the JNK inhibitor SP600125 (JNKi). Samples were collected at the indicated times and examined for total JNK, phosphorylated JNK and caspase-11 by western blot. **(E)** Proposed signaling pathway that regulates inflammasome activation. (A) Data are combined from 9 independent experiments and displayed as the mean ± SEM. (B–D) Data are representative of 3 independent experiments. (n.s. = not significant).

Our data indicate that ROS is capable of regulating caspase-11 expression. However, it remains unclear what pathways are regulated by ROS that subsequently affect caspase-11 expression. As a TLR4-TRIF-IFN-β pathway has been established for expression of caspase-11 [30], [31], we examined the level of type-I interferons, but found they were similar between WT, *Rip2^−/−^*, and *Nod2^−/−^* BMDM (**Figure 6A**). One pathway that is responsive to ROS is the c-Jun N-terminal kinase (JNK) pathway [41]. We next infected WT BMDM with *C. rodentium*, treated them with H_2_O_2_ and examined the effects on JNK1/2 phosphorylation. H_2_O_2_ treatment resulted in increased JNK phosphorylation compared to mock treated cells (**Figure 6B**). Similarly, *Rip2^−/−^* BMDM infected with *C. rodentium* alone had substantially more JNK1/2 phosphorylation than WT cells (**Figure 6C**). Finally, we infected WT or *Rip2^−/−^* BMDM with *C. rodentium* and then treated *Rip2^−/−^* BMDM with the JNK inhibitor SP600125. We observed that caspase-11 levels were reduced in conjunction with reduced JNK1/2 activation (**Figure 6D**). From these results we propose that in the absence of NOD2-RIP2 signaling, defective autophagy results in the accumulation of ROS and the subsequent enhancement of JNK signaling. This ultimately leads to an increase in caspase-11 expression and non-canonical inflammasome activation (**Figure 6E**).

### Increased non-canonical inflammasome activation provides protection against *C. rodentium* induced colitis

**Figure 7:**
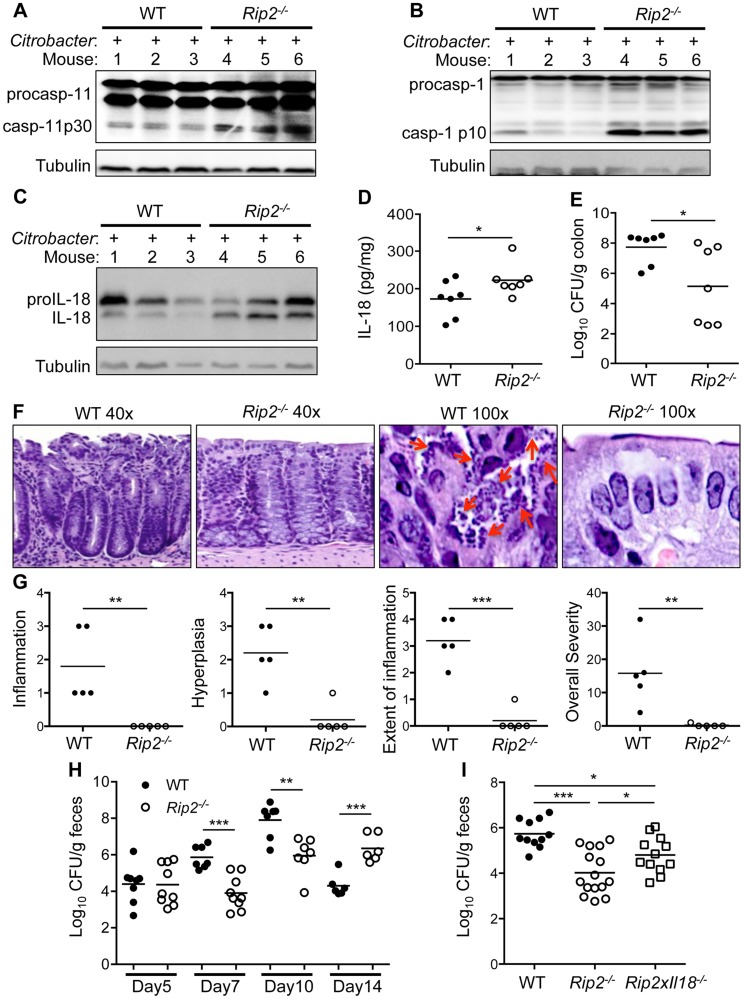
Enhanced inflammasome activation results in protection *in vivo*. WT and *Rip2^−/−^* mice were infected with *C. rodentium*. On day 10 after infection, colon tissue lysates were prepared and analyzed by Western blot for **(A)** caspase-11, **(B)** caspase-1 and **(C)** IL-18. **(D)** IL-18 in colon lysates was also examined by ELISA. **(E)** Colon homogenates were examined for *C. rodentium* colony forming units (CFU) on day 10 after infection. **(F–G)** Colon tissue from WT and *Rip2^−/−^* mice was fixed and H&E stained. (F) Arrows indicate adherent bacteria on the epithelial surface. (G) A veterinary pathologist analyzed H&E stained sections for the indicated clinical scores. **(H–I)** Stool samples from *C. rodentium* infected mice were collected on the indicated days (H) or day 7 (I) and analyzed for *C. rodentium* CFU. (A–H) Data are representative of two independent experiments with n = 6–9 mice per genotype per experiment and displayed as the mean ± SEM. (I) Data are combined from 2 experiments and displayed as the mean ± SEM. (*, p<0.05; **, p<0.01; ***, p<0.001).

To determine the physiological relevance of our proposed pathway, we examined *Rip2*-deficient mice for IL-18 production and inflammasome activation in the colon during *C. rodentium* induced colitis. On day 10 post-infection, caspase-11 and caspase-1 activation were much higher in colon lysates of *Rip2^−/−^* mice compared to WT controls (**Figure 7A,B**). IL-18 activation was significantly increased by Western blot and IL-18 levels were higher in colon lysates taken from *Rip2^−/−^* mice compared to WT controls (**Figure 7C,D**). In contrast, bacterial loads in the colon were reduced in *Rip2^−/−^* versus WT mice (**Figure 7E**). In agreement, colon tissue examined on day 10 after infection showed increased numbers of bacteria adherent to the mucosal surface in WT compared to *Rip2^−/−^* mice (**Figure 7F**). Intriguingly, colon sections from WT mice also showed more hyperplasia and inflammation and the extent of colon area that was inflamed was more pronounced with WT mice compared to *Rip2^−/−^* mice (**Figure 7F,G**). The stool of *Rip2^−/−^* mice also had fewer *C. rodentium* CFU at both day 7 and day 10, highlighting the protective effect of increased inflammasome activation during the innate immune phase of infection (**Figure 7H**). These results demonstrate that the NOD2-RIP2 pathway plays an important role in regulating the non-canonical NLRP3 inflammasome *in vivo* during *C. rodentium*-induced colitis. Of note, previous reports have demonstrated an essential role for adaptive immunity in the eventual clearance of *C. rodentium* [9], [42]. In agreement with these studies, on day 14 after infection, mice deficient in RIP2 signaling had a clearance defect in the stool (**Figure 7H**). Finally, to verify that increased inflammasome activation and IL-18 production were responsible for the lower bacterial numbers on day 7 or 10, we infected WT, *Rip2^−/−^*, and *Rip2^−/−^*×*Il18^−/−^* mice with *C. rodentium* and examined bacterial loads on day 7 after infection. As before, *Rip2^−/−^* mice had significantly lower bacterial numbers compared to WT mice. However, the deletion of IL-18 in *Rip2^−/−^*×*Il18^−/−^* mice resulted in significantly increased bacterial burden compared to the *Rip2^−/−^* mice (**Figure 7I**).

## Discussion

Regulation of the non-canonical NLRP3 inflammasome is still not well understood. It is clear that pathogen entry into the cytosol is required for caspase-11 activation [17], [43], [44]. In addition, caspase-11 activation requires guanylate binding protein (GBP) for activation in response to cytosolic LPS [45]. However, the exact upstream LPS sensor has yet to be identified [46], [47]. Although the exact activation mechanism involved is not clear, our lab and others demonstrated that expression of caspase-11 via a TLR4-TRIF-IFN-β-dependent mechanism is necessary for non-canonical NLRP3 inflammasome activation during *C. rodentium* infection [30], [31]. Our current findings show that ROS produced during *C. rodentium* infection, or added exogenously to infected cells as H_2_O_2_, is capable of regulating caspase-11 expression. In agreement, treatment with the ROS scavenger NAC dampened caspase-11 expression and activation, and subsequently reduced caspase-1 activation through the non-canonical NLRP3 inflammasome. Mechanistically, *Rip2^−/−^* cells display enhanced ROS production following *C. rodentium* infection, and our data define a pathway where ROS acts through JNK signaling to increase caspase-11 expression and the subsequent activation of the non-canonical NLRP3 inflammasome.

Similar to *C. rodentium*, we found that infection with flagellin-deficient *S. typhimurium Δfljb/flic* resulted in augmented inflammasome activation in *Nod2^−/−^* and *Rip2^−/−^* BMDM. Since activation of the NLRC4 inflammasome was comparable between WT and *Rip2*-deficient BMDM infected with WT *S. typhimurium*, these data suggest that NOD2-RIP2 mediated regulation of ROS and JNK activation specifically modulates the non-canonical NLRP3 inflammasome and not NLRC4.

In addition to the mechanistic insight provided by our *in vitro* studies, caspase-1 activation has never been examined in *Rip2^−/−^* mice in response to *C. rodentium*. Our findings demonstrate the NOD2-RIP2 pathway helps suppress the non-canonical NLRP3 inflammasome *in vivo* during *C. rodentium* induced colitis. In the absence of RIP2, there is increased caspase-11 and caspase-1 activation and higher levels of processed IL-18. These increases are associated with reduced bacterial burden and protection of the colon epithelium during the innate immune phase of infection (d7–d10). Our findings are thus in line with our previous publications, which show that NLRP3 inflammasome activation and IL-18 production are important for the control of bacterial burden and disease during *C. rodentium* infection [48]. The importance of IL-18 in the protection seen in this model was also confirmed through the use of *Rip2^−/−^*×*Il18^−/−^* mice. Although IL-18 deletion in *Rip2^−/−^* mice did not entirely abolish the protective phenotype, this is likely due to pyroptosis or other functions of caspase-11, such as lysosome-phagosome fusion [49].

Other reports have examined NOD2 signaling *in vivo*. However, these reports focused on the effects of NOD2-RIP2 signaling on the adaptive immune response. Intriguingly, similar results to ours were published for the ubiquitin ligase pellino3, which is required for NOD2-RIP2 signaling. At early time points, *Peli3^−/−^* mice had reduced bacterial burden similar to our model, but after d14, these mice showed a clearance defect [42]. On the other hand, *Nod2^−/−^* mice displayed reduced production of the chemokine CCL2, which ultimately lead to impaired adaptive immunity and impaired clearance of *C. rodentium* after d14 of infection [9]. Collectively, these findings suggest that increased NLRP3 inflammasome activation provides protection early during the innate immune phase, but the defects in adaptive immunity that are also present in *Nod2^−/−^, Rip2^−/−^, or Peli3^−/−^* mice ultimately lead to an inability of these mice to resolve the infection.

Although inflammation and cell death are important for bacterial clearance [25], they also increase the likelihood of bacterial dissemination to other tissues and induction of severe inflammatory responses by increased tissue damage and released cellular contents [50]–[52]. Therefore, the sequential and carefully orchestrated activation of inflammatory pathways is essential for pathogen clearance as well as maintaining homeostasis in the host. Initial recognition of specific peptidoglycan fragments from bacterial cell walls by the NOD2-RIP2 pathway, results in pro-inflammatory NF-κB and MAP-kinase activation within minutes of infection [53], [54]. However, if this initial inflammatory burst is inadequate, then subsequent inflammatory pathways, such as the non-canonical NLRP3 inflammasome, are initiated. One common feature of pathogens that activate the non-canonical NLRP3 inflammasome is activation proceeds with slower kinetics than during canonical activation. In all instances, non-canonical NLRP3 inflammasome activation is observed only after a time-period of 12 to 16 h [26], [28], [30], [31], whereas canonical activation requires less than 1 h [21], [22], [55]. Based on our results, we propose that NOD2-RIP2 signaling initially suppresses or delays non-canonical NLRP3 inflammasome activation by preventing or removing mitochondrial damage. However, extended assault from *C. rodentium* eventually overcomes these mechanisms and leads to ROS generation and activation of the non-canonical NLRP3 inflammasome [20], [39], [56]. In the absence of NOD2 or RIP2, increased mitochondrial damage and ROS production leads to elevated JNK activation, and in turn to augmented non-canonical NLRP3 inflammasome activation. During *C. rodentium* infection *in vivo*, this impaired initial bacterial colonization of the colon and provided protection of the epithelium up to days 10 after infection. However, in other colitis models, increased inflammasome activation may result in increased damage to the colon tissue if left unchecked. The NOD2-RIP2 pathway has been associated with Crohn's disease in humans [57] and plays an essential role in several mouse models of colitis [58]–[62]. As *Rip2*-deficiency leads to enhanced inflammasome activation, it will be of interest to examine caspase-1 activation in other models where inflammation is a key factor and where the NOD2-RIP2 axis is involved.

## Materials and Methods

### Ethics statement

Experiments were conducted under protocols approved by the St. Jude Children's Research Hospital Committee on Use and Care of Animals (Protocol #482) and were performed in accordance with institutional policies, AAALAC guidelines, the AVMA Guidelines on Euthanasia (CO_2_ asphyxiation followed by cervical dislocation), NIH regulations (Guide for the Care and Use of Laboratory Animals), and the United States Animal Welfare Act (1966).

### Mice

All mice were maintained at SJCRH and were backcrossed for at least 10 generations onto the C57BL/6J (B6) background. *Nod2^−/−^*, *Rip2^−/−^* and *Rip2^−/−^*×*Il18^−/−^* mice have been reported previously [39], [63], [64]. WT-GFP-LC3^+^ and *Rip2^−/−^*-GFP-LC3^+^ mice were generated by crossing WT or *Rip2^−/−^* mice with LysM-Cre^+^ *Atg7^loxP/loxP^* GFP-LC3^+^ mice [39] and selecting progeny that only contained the GFP-LC3 transgene but not LysM-Cre or *Atg7^loxP/loxP^*. All mice were housed in the SJCRH animal resource center, which is a specific pathogen free (SPF) and AAALAC accredited facility.

### Bacterial infection, caspase-1 activation and IL-18 production

WT, *Nod2* ^−/−^ and *Rip2* ^−/−^ bone marrow derived macrophages (BMDM) were differentiated in complete IMDM containing 10% heat-inactivated FBS and supplemented with M-CSF containing L929 supernatant at 37°C in a humidified atmosphere containing 5% CO2 for 5 days. *Citrobacter rodentium* (ATCC 51459), *Salmonella typhimurium* (SL1344), *Salmonella typhimurium* Δ*fljb/flic* were grown in LB broth overnight with shaking at 37°C. Next day, the bacteria were subcultured to mid-log phase, washed in PBS and BMDM were infected with 20 multiplicity of infection (MOI) of *C. rodentium*, 1MOI *of S. typhimurium*, or 10MOI of *S. typhimurium* Δ*fljb/flic*. Cell lysates combined with supernatants were collected after 18 h of infection and assessed for caspase-1 activation by Western blot using the anti-caspase-1 p20 antibody from Adipogen (AG-20B-0042-C100). Western blots were imaged on a Kodak IMM4000pro and the densitometry performed using the Carestream software provided by the manufacturer. Caspase-1 p20 levels were normalized to the proCaspase-1 for that lane. Supernatants collected at 18 h after infection were analyzed for IL-6, TNF-α, or IL-18 by ELISA according to the manufacturer's instructions (eBiosciences) or for type-I interferon by the RAW-Blue ISG cell line (Invivogen).

### *C. rodentium* growth assay in BMDM

WT, *Nod2^−/−^* and *Rip2^−/−^* macrophages were grown in 24-well plates and infected with 10 MOI of *C. rodentium* at 37°C. After 2 h of infection, cells were washed three times with PBS and 10 µg/ml of gentamicin was added to kill extracellular bacteria. Cells were lysed in 0.1% Triton X-100 at indicated times and lysates were serially diluted and plated on LB agar plates. The colonies were counted after 18 h of growth at 37°C

### Treatments

For SP600125 (JNK inhibitor, 10 µM, Santa Cruz Biotechnology), N-acetyl-L-cysteine (NAC, 10 mM, Sigma) or glyburide (200 µM, Sigma) treatment, BMDMs were exposed to these inhibitors after 2 h of *C. rodentium* infection. For H_2_O_2_ treatment, BMDM were treated at 6 h after infection. For MG-132 (1 µM) treatment, BMDM were treated at 4 h after infection, at 2, 4, 8 or 18 h after infection, supernatants were collected for ELISA and cells or cells + supernatants were lysed with RIPA buffer containing protease inhibitors and phosphatase inhibitors (Calbiochem), followed by boiling in sodium dodecyl sulfate (SDS) sample buffer and examined by Western blot. Anti-caspase-1 p20 (Adipogen), anti-caspase-1 p10 (Santa Cruz Biotechnology, A20), anti-total JNK (Cell signaling Technologies, 9252), anti-phospho JNK (Cell signaling Technologies, 9251L) and anti-caspase-11 antibodies (Enzo, 4E11) were used for Western blot detection and equal loading was verified by blotting with anti-GAPDH or anti-Tubulin (Cell Signaling Technologies). HRP-labeled anti-mouse or -rabbit antibodies were obtained from Jackson Immuno Research. Caspase-11 densitometry was normalized to the procaspase-11 level in the uninfected lane for each sample. Phospho-JNK densitometry was normalized to GAPDH loading control for each lane. IL-18 levels were determined in the supernatants by ELISA according to manufacturer's instructions (eBioscience). Alternatively, cells were collected at the indicated time points and RNA isolated for analysis by qRT-PCR. Primers: GAPDH forward 5′-CGTCCCGTAGACAAAATGGT-3′ reverse 5′-TTGATGGCAACAATCTCCAC-3′; IL-18 forward 5′-GCCTCAAACCTTCCAAATCA-3′ reverse 5′-TGGATCCATTTCCTCAAAGG-3′; Caspase-11 forward 5′-ACAATGCTGAACGCAGTGAC-3′ reverse 5′-CTGGTTCCTCCATTTCCAGA-3′.

### ROS production

For staining mitochondria, uninfected BMDMs were used as controls or infected with 20MOI of *C. rodentium* for 18 h and then stained for 30 min in fresh medium containing 5 µM MitoSOX. Cells were then washed in HBSS, resuspended in flow cytometry buffer (DPBS with 1% FBS and 0.04% NaN3) and analyzed immediately by flow cytometry.

### Autophagy

WT-GFP-LC3^+^ and *Rip2^−/−^*-GFP-LC3^+^ mice were used to generate BMDM as before and cells were infected with 20MOI of *C. rodentium* for 4 h or 18 h. Cells were then permeabilized with 0.05% saponin in PBS for 5 min to remove cytosolic GFP-LC3 and cells were fixed in 4% paraformaldehyde in PBS for 5 min. Cells were then examined by fluorescence confocal microscopy for the number of GFP-LC3^+^ puncta after staining with Alexafluor 647 phalloidin and mounting with ProLong Gold+DAPI (Life Technologies). Alternatively, cell were scraped from wells and resuspended in FACS buffer and examined by flow cytometry for GFP intensity as a readout of autophagy.

### *Citrobacter rodentium* induced colitis

Age and sex matched 8–10 week old mice were infected with 10^10^ CFU of *C. rodentium* by oral gavage. Stool was collected from mice for CFU at the indicated time points. Mice were euthanized on D10 and colons collected for CFU determination by homogenizing in PBS or for Western blot by homogenizing in RIPA buffer containing protease inhibitors and phosphatase inhibitors (Calbiochem). Protein concentration was normalized and samples analyzed by western blot; anti-IL-18 (MBL, 39-3F) anti-caspase-1 p10 (Santa Cruz Biotechnology, A20) and anti-caspase-11 antibodies (Enzo, 4E11) were used. Lysates were additionally examined by ELISA for IL-18 (Ebiosciences) and normalized to total protein. D10 colon samples were also collected, formalin fixed and processed for routine histopathological examination by hematoxylin and eosin staining. Sections were examined by a board certified veterinary pathologist (Peter Vogel) for inflammation, bacterial invasion of epithelial surface, and distribution of inflammation.

### Statistics

All statistical analyses were performed using GraphPad Prisim 6.0. Students *t*-Test was used for single comparisons and One-Way ANOVA with Dunnett's or Sidak's post-hoc test for multiple comparisons. Two-way ANOVA was used to analyze *C. rodentium* growth between groups over time.
